# Provider reported value and use of virtual resources in extended primary care prior to and during COVID-19

**DOI:** 10.1186/s12913-022-08752-2

**Published:** 2022-11-15

**Authors:** Jolie N. Haun, Vanessa Panaite, Bridget A. Cotner, Christine Melillo, Hari H. Venkatachalam, Christopher A. Fowler, Brian Zilka, William Messina

**Affiliations:** 1 Research and Development Service, James A. Haley Veterans’ Hospital and Clinics, 8900 Grand Oak Circle, Tampa, FL 33637 USA; 2 College of Public Health, University of South Florida, 4202 E. Fowler Avenue, Tampa, FL 33620 USA; 3 Department of Psychology, University of South Florida, 4202 E. Fowler Avenue, Tampa, FL 33620 USA; 4 Department of Anthropology, University of South Florida, 4202 E. Fowler Avenue, Tampa, FL 33620 USA; 5 Department of Psychiatry and Behavioral Neurosciences, University of South Florida, 4202 E. Fowler Avenue, Tampa, FL 33620 USA; 6 Primary Care, James A. Haley Veterans’ Hospital and Clinics, 13000 Bruce B. Downs Boulevard, Tampa, FL 33612 USA; 7 Ambulatory Care, James A. Haley Veterans’ Hospital and Clinics, 13000 Bruce B. Downs Boulevard, Tampa, FL 33612 USA

**Keywords:** Primary care, Technology, Veteran, Virtual healthcare resources

## Abstract

**Background:**

A proactive approach to delivering care using virtual resources, while reducing in-person contact, is needed during the COVID-19 pandemic.

**Objective:**

In the current study we describe pre- to post- COVID-19 pandemic onset related changes in electronic delivery of primary care.

**Methods:**

A longitudinal, pre-post within-subjects design was used. Patient-aligned care team providers from one VA medical center, a primary care annex, and four affiliated community-based outpatient clinics completed both a baseline and follow up survey (*N* = 62) or the follow-up survey only (*N* = 85). The follow-up survey contained questions about COVID-19.

**Results:**

The majority of providers (88%) reported they would continue virtual care once pandemic restrictions were lifted. Most (83%) felt prepared to transition to virtual care when pandemic restrictions began. Use of My Health***e***Vet, Telehealth, and mobile apps showed a significant increase (22.7%; 31.1%; 48.5%). Barriers to virtual care included (1) internet connectivity; (2) patients’ lack of technology comfort and skills; and (3) technical issues. Main supports to provide virtual care to patients were (1) peers/ colleagues; (2) technology support through help desk; (3) equipment such as laptops and dual screens; (4) being able to use doximety and virtual care manager, and (5) training.

**Conclusions:**

Overall, provider-use and perceptions related to using virtual care improved over time. Providers adapted quickly to providing virtual care during COVID-19 and planned to provide virtual care long-term.

## Introduction

Historically, virtual healthcare resources (VHR) have been used to enhance patient access to healthcare, especially in rural areas [1]. The Department of Veterans Affairs (VA) has an enterprise-wide effort to improve access to patient-centered care using VHR. VA’s effort to promote use of VHR includes an extensive suite of resources (Table 1). Though the VA has an ongoing virtual care delivery initiative, the COVID-19 pandemic required healthcare systems to use VHR to expand access to care while reducing in-person contact [2, 3].

**Table 1:** Virtual healthcare resources

Virtual Healthcare Resources	Description	Patient-facing	Provider-facing
My Health***e***Vet			
Appointment Tool	Appointment module of My Health***e***Vet suite that allows patients to request, review and cancel appointments.		
Blue Button	Medical record module of My Health***e***Vet suite that allows patients to view, download and print selected self-entered and VA Electronic Health Record (EHR) information.		
Healthy Living Assessments	Assessment module of My Health***e***Vet suite that identifies health age and offers suggestions to improve health age (e.g., smoking cessation, weight loss).		
Journals	Journaling module of My Health***e***Vet suite offers place to document food intake and activities.		
Labs and Tests	Report module of My Health***e***Vet suite that allows patients to enter test results and provides tables and graphs of VA-generated lab and test results		
Rx Refill	Prescription refill module of My Health***e***Vet suite. Can be accessed via internet or mobile app.		
Secure Messaging	Secure Messaging feature of My Health***e***Vet suite.		
Veterans’ Health Library	Electronic library module of My Health***e***Vet suite that provides educational content by condition (e.g., asthma, diabetes).		
Vitals Tracker	Data entry and report module of My Health***e***Vet suite that provides graphs of patient-entered vital signs (e.g., blood pressure, weight) over time.		
Mobile Apps			
Mobile apps-VA (https://mobile.va.gov/appstore*)*	Variety of VA developed applications that can be used for patient self- management, in conjunction with a VA therapy (e.g., smoking cessation, mindfulness, chronic condition management) or by providers to facilitate care management.		
Mobile apps- Non-VA	Variety of electronic applications which target specific needs (e.g., smoking cessation, mindfulness, chronic condition education) and are not sponsored by nor vetted through the VA.		
Telehealth			
Telehealth-VA Video connect	Real-time communication between patient and provider used to gather physical assessment data and provide one on one communication between patient and provider.		
Telehealth-Asynchronous	A variety of communication technologies that provide asynchronous communication (e.g., an image is sent to a remote provider who then reviews and acts upon it).		
Telehealth-Care Coordination Home	Remote monitoring of Veterans who require intensive monitoring by nurses who check in with them via telephone several times a week.		
Telephone	Device (land line or mobile) that provides live audio communication between patient and provider.		
VetLink Kiosks	VA electronic device which patients use to check into appointments and update personal and insurance information.		

VA has spent the last decade improving patient-centered care through promotion of proactive integrated use of VHR, specifically within primary care. Proactive integrated VHR use is defined as ‘a self-initiated approach to coordinated use of applicable VHR systems for the purposes of coordinating and delivering timely high-quality patient-centered care’ [4]. Provider openness to use VHR was promoted by system preparedness (e.g., IT systems, software, policies) and the necessity brought on by COVID-19 pandemic. For example, between mid-March and late April 2020, the number of VA weekly video-to-home primary care encounters rose over 11-fold and the number of clinicians using video-to-home visits increased 22% [5]. Telephone visits increased by 131% [5].

In this study we evaluated pre- to post-COVID-19-related changes in delivery of primary care at one VA medical center and associated sites (VAMC). “Post-COVID-19” refers to changes in care delivery put into place in response to the pandemic. We conducted a survey of VHR use and perceptions among primary care providers [aka, Patient Aligned Care Teams (PACT)] prior to onset of the pandemic (January 2020) and after COVID-19-related changes to care (October 2020). Understanding the use of VHR before and during the pandemic will provide a baseline of VHR use while acknowledging the influence of the pandemic. This data will allow for long-term assessment of VHR use and inform opportunities to continue VHR use over time.

## Methods

### Parent study

The parent study [6] was designed to evaluate implementation strategies to promote VHR use among primary care providers. After consent, data were collected via paper and online surveys through VA Research Electronic Data Capture (REDCap) [7, 8]. Dillman method [9] was used to increase response rates. Analyses comparing providers who received VHR training (*n* = 45) versus those who did not (*n* = 17) on demographics, baseline, and follow-up survey data resulted in no significant group differences (*p* > .05). Differences in providers’ use and perceived benefits of VHR are attributed to the onset of the COVID-19 pandemic which provided an unexpected opportunity to evaluate experiences of VHR use during widespread disruption of the healthcare system. This research protocol was reviewed and approved by the University of South Florida Institutional Research Board. Informed consent was obtained according to University of South Florida Institutional Research Board guidance.

### Design

The current study used a pre-post within-subjects design. Data were collected from providers at two time points: January 2020 and October 2020. Consolidated Framework for Implementation Research (CFIR) [10] constructs were used to guide data collection and analysis.

### Sampling

We recruited PACT providers from one VAMC. Sixty-two providers completed baseline and follow-up surveys; an additional 23 providers completed the follow-up survey only, for a total of 85 providers completing COVID-19 questions in the follow-up survey. A priori power analysis indicated a sample size of 63 to achieve 86% power to detect a medium effect size (Cohen’s *d* = .5). An evaluation of study attrition indicated no demographic differences (*p > .05*) between participants who completed a post survey (*n* = 62) and those missing follow-up data (*n* = 47).

### Measures

A 16-item questionnaire assessed demographics; an 18-item self-report survey, previously described [6], was organized into 5 subscales informed by CFIR constructs, including *relative advantage* (perceptions of improved care delivery, preference over traditional tools); *observability* (perceptions of improved clinical workflow and patient outcomes); *compatibility* (self-reported comfort with VHR use to communicate and deliver care); *complexity* (education is needed on access/use, integration ease in patient care delivery); *context and facilitation* (perceptions of VHR use reflects role responsibilities and is reinforced by workplace) [11]. Items were scored on a 5-point Likert scale (1 = strongly agree to 5 = strongly disagree) that were averaged at the subscale level. The follow-up survey included nine additional questions related to COVID-19: six regarding use of VHR pre, during, and post (projected) and three open-ended about barriers, supports, and preference for providing virtual care. All questions are aimed at capturing provider perspective of their own and their patient’s use and change in use of VA resources. VHR listed in the survey are described in Table 1.

### Analysis

Descriptive statistics summarize sample demographics. Given the non-independent nature of our within-group sample, we used the following analyses to evaluate pre- to post-changes: 1) nonparametric McNemar’s test to evaluate VHR utilization pre- to post-COVID-19 (Table 3); 2) paired sample *t*-tests to evaluate changes in provider perceptions of patients’ preferred methods of communication, and promotion of VHR with their patients (Table 4); and 3)Cohen’s *d* effect sizes with 95% confidence intervals are reported in Table 4. Rapid matrix analysis [12] and content analysis [13] was used to analyze the three open-ended questions.

## Results

### Provider characteristics

Participants with pre and post surveys (*N* = 62) represented all PACT roles; nearly half representing nurses (45.2%). The sample was predominately white (77.4%), female (85.5%), with an average age of 46.2 years (Table 2).

**Table 2:** Primary care participant baseline characteristic

	Total (***N*** = 62)
**Characteristic**	N (%)
**Role**	
Provider	14 (22.6)
Nurse	28 (45.2)
Clinical Associate (LPN)	13 (21.0)
Other (Pharmacists, Psychologists, Dietician, Mental Health Nurse, Whole Health)	7 (11.3)
**Race (all that apply)**	
Black, African American	5 (8.1)
Asian (Chinese, Filipino, Japanese, Korean etc.)	3 (4.8)
White, Caucasian	48 (77.4)
Native Hawaiian or other Pacific Islander	1 (1.6)
American Indian or Alaskan Native	1 (1.6)
Unknown	1 (1.6)
Other (Hispanic, Hispanic white, mixed)	3 (4.8)
Declined to respond	1 (1.6)
**Ethnicity**	
Hispanic	12 (19.4)
Not Hispanic	44 (71.0)
Decline to Respond	2 (3.2)
**Gender**	
Female	53 (85.5)
**Age (mean, SD)**	46.2 (11.0)

### Quantitative findings

#### Provider reported virtual practice pre, during, and projected post COVID-19

Of the follow-up participants (*N* = 85, see Table 2), 75% reported 25% or less of their care delivery was conducted virtually prior to COVID-19. Only 1% of providers reported 25% or less of their practice was conducted virtually during COVID-19. When asked to predict whether their practice would continue virtually after COVID-19, 48% predicted less than half of their care being delivered virtually versus 52% predicting more than half of their care being delivered virtually; 88% reported, if possible, they would continue conducting care virtually once COVID-19-related restrictions are lifted. Majority (83%) reported feeling prepared to implement COVID-19-related changes to care. Finally, 39% reported information received during the parent study training helped respond to COVID-19 changes to care.

#### Changes in provider perception of their and patients’ use and promotion of VHR

While use of Secure Messaging remained elevated over the study period with over 87% of providers reporting use, survey results highlighted changes in use of My Health***e***Vet, Telehealth VA Video Connect (referred to as Telehealth hereafter), VetLink Kiosks, and mobile apps (Table 3). Despite these changes, providers reported patient preference for most VHR remained stable. In contrast, patient preference for Telehealth doubled (Table 3).

**Table 3:** Baseline and six-month follow-up use and promotion of virtual medical modalities (virtual resources) among PACT providers (*N* = 62)

	Baseline	Six-month Follow-up	Percent Change	McNemar Exact Test
	**Provider’s virtual resources use (% yes)**			
My Health***e***Vet	66.1	85.5	29.4%	.004**
Secure Messaging	88.7	87.1	−1.8%	1.00
Telehealth	50.0	72.6	45.2%	.007**
VetLink Kiosks	53.2	17.7	− 66.7%	<.001***
Mobile apps	27.4	53.2	94.2%	.002**
	**Provider perception of patients’ preferred methods of communication (% yes)**			
Telephone	82.3	75.8	−7.9%	.424
Face-to-face	79.0	69.4	−12.2%	.146
My Health***e***Vet	38.7	32.3	−16.5%	.541
Secure Messaging	82.3	87.1	5.83%	.581
Telehealth	17.7	38.7	118.6%	.019*
VetLink Kiosks	11.3	4.8	− 57.5%	.289
Mobile apps	11.3	12.9	14.2%	1.00
	**Providers’ promotion of patients’ use of virtual resources (% yes)**			
My Health***e***Vet	87.1	90.3	3.7%	.754
Secure Messaging	96.8	93.5	−3.4%	.687
Telehealth	61.3	72.6	18.4%	.230
VetLink Kiosks	45.2	33.9	−25.0%	.189
Mobile apps	30.6	41.9	36.9%	.189
	**Promotion of patients’ use of virtual resources on behalf of providers (% yes)**			
My Health***e***Vet	5.1	3.4	−33.3%	1.00
Secure Messaging	6.6	1.6	−75.8%	.375
Telehealth	5.3	3.5	− 34.0%	1.00
VetLink Kiosks	5.3	12.3	132.1%	.289
Mobile apps	3.9	13.7	251.3%	.063
	**% Patients with whom providers report use/promote virtual resources (% responded 50–100%)**			
My Health***e***Vet	75.0	78.6	4.8%	.815
Secure Messaging	79.3	84.5	6.6%	.607
Telehealth	40.4	84.6	109.4%	<.001***
VetLink Kiosks	64.3	59.5	−7.5%	.791
Mobile apps	28.6	50.0	74.8%	.049

***p < .001, **p < .01, *p < .05

Telehealth = VA Video Connect

Similarly, provider promotion of patient use and their own use of VHR remained stable, with over 75% reporting continued use or promoting My Health***e***Vet and Secure Messaging with 50–100% of their patients. However, providers’ use of mobile apps and Telehealth increased (Table 3).

#### Changes in provider perceptions

Providers’ perceptions of *relative advantage* for use of My Health***e***Vet, Secure Messaging, and Kiosks were stable (i.e. small effect sizes) showing providers continued to agree VHR use improved care delivery and was preferred over other tools (e.g., telephone). The two VHR that showed improvements in provider perceptions were Telehealth and mobile apps (Table 4).

**Table 4:** Perceptions about virtual resources among providers (*N* = 62) at baseline and six-month follow-up

	Baseline	Six-month Follow-up	***Paired sample t-test***	***p***	***Cohen’s d (95%CI)***
**Relative advantage** (improved care delivery, preference over traditional tools) **(M, SD)**					
My Health***e***Vet	1.61 (.90)	1.55 (.71)	0.45	.657	0.07 (−0.28,0.43)
Secure Messaging	1.62 (.93)	1.45 (.58)	1.20	.237	0.22 (−0.13,0.57)
Telehealth	1.95 (1.04)	1.63 (.69)	1.97	.054	0.36 (0.01,0.72)
VetLink Kiosks	2.42 (1.53)	2.43 (1.28)	−0.08	.937	0.01 (− 0.34,0.36)
Mobile apps	2.72 (1.45)	2.19 (1.14)	2.45	.017*	0.41 (0.05,0.76)
**Observability** (improved clinical workflow and patient outcomes) **(M, SD)**					
My Health***e***Vet	1.75 (.83)	1.52 (.66)	2.26	.028*	0.31 (−0.05,0.66)
Secure Messaging	1.70 (.86)	1.46 (.53)	2.16	.035*	0.34 (−0.02,0.69)
Telehealth	2.20 (1.15)	1.54 (.65)	3.77	<.001***	0.71 (0.34,1.07)
VetLink Kiosks	2.53 (1.58)	2.42 (1.30)	0.60	.549	0.08 (− 0.28,0.43)
Mobile apps	2.92 (1.51)	2.28 (1.23)	3.04	.004**	0.46 (0.11,0.82)
**Compatibility** (comfort with virtual resources use to communicate and deliver care) **(M, SD)**					
My Health***e***Vet	1.86 (1.17)	1.46 (.87)	2.62	.011*	0.39 (0.03,0.74)
Secure Messaging	1.48 (.95)	1.25 (.57)	1.75	.085	0.29 (−0.06,0.65)
Telehealth	2.45 (1.70)	1.48 (1.01)	4.50	<.001***	0.69 (0.33,1.05)
VetLink Kiosks	3.07 (1.98)	2.78 (1.56)	1.14	.261	0.16 (−0.19,0.52)
Mobile apps	3.17 (1.82)	2.42 (1.52)	2.93	.005**	0.45 (0.09,0.80)
**Complexity** (education is needed on access/use, integration ease in patient care delivery) **(M, SD)**					
My Health***e***Vet	1.80 (.80)	1.66 (.81)	1.55	.126	0.17 (−0.18,0.53)
Secure Messaging	1.67 (.72)	1.59 (.73)	0.91	.366	0.11 (−0.24,0.46)
Telehealth	2.19 (.99)	1.63 (.69)	4.50	<.001***	0.66 (0.29,1.02)
VetLink Kiosks	2.45 (1.33)	2.46 (1.23)	−0.04	.967	0.01 (− 0.34,0.36)
Mobile apps	2.35 (1.04)	2.16 (1.01)	1.35	.182	0.19 (− 0.17,0.54)
**Context and facilitation** (use reflects role responsibilities, reinforced by workplace) **(M, SD)**					
My Health***e***Vet	1.70 (.82)	1.60 (.84)	0.95	.344	0.12 (−0.23,0.47)
Secure Messaging	1.46 (.51)	1.40 (.47)	0.77	.442	0.12 (−0.23,0.47)
Telehealth	1.84 (.97)	1.46 (.58)	3.04	.003**	0.48 (0.12,0.83)
VetLink Kiosks	2.33 (1.48)	2.58 (1.49)	−1.08	.283	0.17 (−0.18,0.52)
Mobile apps	2.45 (1.40)	2.35 (1.31)	0.47	.638	0.07 (−0.28,0.43)

*CI* = Confidence interval, *M* = mean, *SD* = Standard deviation

***p < .001, **p < .01, *p < .05

Telehealth = VA Video Connect

Two domains that showed moderate to large effect size changes were *perceptions of observability* and *compatibility*. Providers reported a moderate effect size increase in perceived observability and compatibility for use of My Health***e***Vet, a moderate to large effect size increase for use of mobile apps, and a large effect size increase for use of Telehealth (Table 4).

Telehealth was the only VHR that showed large increases in perceived *complexity* and *context and facilitation* with its use, with other VHR showing stability over time (Table 4). Providers were more likely to report at follow-up that education is needed in using Telehealth and integrating it into care.

### Qualitative findings

#### Provider reported barriers, facilitators, and changes in VHR preferences

Provider identified main barriers were (1) internet connectivity; (2) patients’ lack of skills using technology; and (3) technical issues. Main supports to provide virtual care to patients were (1) peers/colleagues; (2) technology support through help desk; (3) equipment to support virtual care delivery (e.g., laptops); (4) online networking services, and (5) training. Half of the providers (48%) appreciated virtual care more or were more open to it. Some reasons cited for this preference were improved: confidence, convenience, workflow, and continuity of care.

## Discussion

In this study we described changes in primary care delivery pre- to post- COVID-19 pandemic onset practice changes at one VAMC and affiliated sites. We conducted a survey of VHR utilization and perceptions among PACT members in January 2020 and after pandemic-related changes to care were underway (October 2020). Data representing VHR use before and during the pandemic will provide a baseline of VHR use and inform opportunities to sustain VHR use over time. Our study is unique in that it is one of the first data samples to describe providers’ VHR use and perceptions within the VA both before and after COVID-19 changes in care delivery. These data provide a baseline study for VHR use changes and can inform use patterns over time.

While use of Secure Messaging remained consistent from pre- to post-evaluation, results highlighted changes in use of other tools (e.g., My Health***e***Vet, mobile apps). These changes are consistent with transition to mainly virtual delivery in March 2020. We recorded an increase in VHR designed for remote care, such as apps, paired with an expected decrease in tools associated with face-to-face visits such as VetLink Kiosks. These findings are consistent with data we collected on provider reports of pre- to post- delivery of care changes in their own practices.

We also found providers’ perceptions of patient preferences changed. Providers reported nearly twice as many patients preferred Telehealth to face-to-face visits. Although we only collected providers’ perceived patient preference in this study, this work was an extension of the authors’ previous works, which focused on patient perspective and experiences with VHR [14–18]. The authors focus on providers’ perspective was driven by patient reports that providers’ recommendations and reinforcement of VHR use drive patient use patterns [17]. Findings align with previous work suggesting patients perceived managing their care with VHR alleviated care burden by improving access to knowledge and care, and improving continuity and experience of care [16, 19]. Findings indicate provider promotion of patient use and their own use of VHR predates COVID-19. Providers and patients were familiar with the tools and primed to implement care changes, which translated to doubling promotion and use of telehealth from pre- to post- COVID-19.

Overall, provider positive perceptions regarding VHR use remained stable over time. Providers reported perceived benefits and preference for using mobile apps to deliver care; and were more likely to agree that use of My Health***e***Vet, mobile apps, and telehealth was associated with improved clinical workflow, patient outcomes, and workflow at follow-up. Telehealth was the only VHR showing large increases in perceptions that further education is needed to integrate telehealth into care delivery. These findings suggest having access to a wide array of VHR can improve workflow by more flexibly responding to needs; however, providers would desire more training, given the recent rapid shift in using VHR to deliver healthcare [5].

Our COVID-19-related questions shed light on providers’ experiences with adapting to COVID-19-related care changes. Most of the sample reported feeling prepared to implement VHR during COVID-19. This is likely in part due to belonging to a system that had made efforts well before COVID-19 to implement VHR for care delivery. This preparedness could be related to the parent study’s training provided to PACT in the month prior to COVID-19-related changes. Training and targeted implementation strategies are needed to support providers’ proactive integrated VHR use while providing consistently high-quality care [2]. Qualitative findings suggested providers’ preference for virtual care for most types of visits. Finally, the experience of adapting to COVID-19-related changes in delivery of care while abrupt, is likely to have lasting effects, with more than three-fourths of providers reporting desire to continue conducting care virtually once restrictions are lifted. Sustainability of VHR use is needed beyond the recent COVID-19 pandemic context. Future work should assess sustainability of VHR use and quality of patient experiences and outcomes. Previous efforts such as mandates and workload credit were successfully implemented to support uptake [14] and sustained use over time.

Study limitations should be considered when interpreting findings, including the use of self-report, sample size, and the unique VA environment. Authors did not evaluate the medicolegal implications of the usage of VHRs post pandemic as that is beyond the scope of this study. Such evaluation should be addressed in future work as global pandemics and the need for VHR use are likely to remain relevant to health services. These data provide insights into VHR use in an unprecedented context and can be used to inform a subsequent study using a larger sample to assess convergence with these findings. Future research is also warranted to determine if VHR user trends persist post-pandemic, and if PACT providers – and patients – need education to maximize the opportunity to proactively integrate VHR into the healthcare delivery process.

## Conclusions

Data indicate providers were prepared to use VHR within their practice, yet many did not embrace implementation of proactive integrated use of VHR prior to COVID-19 related care delivery changes. The pandemic necessitated implementation of proactive use of VHR. Providers reported increased use, improved perceptions, and intention to continue use of VHR, consistent with the VA system wide effort to promote the delivery of care virtually. Future research is needed to build upon the current findings to determine if VHR use sustains post pandemic and assess quality of patient experiences and outcomes.

## Data Availability

The datasets during and/or analyzed during the current study available from the corresponding author on reasonable request.
